# Protective effects of *Lactobacillus plantarum* strain against protein malnutrition-induced muscle atrophy and bone loss in juvenile mice

**DOI:** 10.1371/journal.pone.0317197

**Published:** 2025-01-16

**Authors:** Hyerim Park, Sung-Hee Kim, Kyung-Ah Lee

**Affiliations:** School of Biological Sciences, Seoul National University, Seoul, South Korea; Fujita Health University, JAPAN

## Abstract

Early-life malnutrition adversely affects nearly all organ systems, resulting in multiple physiological adaptations, including growth restriction and muscle and bone loss. Although there is growing evidence that probiotics effectively improve systemic growth under malnourished conditions in different animal models, our knowledge of the beneficial effects of probiotics on various organs is limited. Here, we show that *Lactobacillus plantarum* strain WJL (Lp^WJL^) can mitigate skeletal muscle and bone loss in protein-malnourished juvenile mice. Mice on prenatal day 21 were fed a protein-malnourished (P-MAL) diet with or without Lp^WJL^ supplementation for six weeks. Compared to mice on the P-MAL diet alone, Lp^WJL^ supplementation significantly increased muscle mass and size, resulting in enhanced muscle strength and endurance capacity. Furthermore, Lp^WJL^ supplementation induced the expression of the key growth factor IGF-1 while decreasing muscle atrophy markers such as Atrogin-1 and MuRF-1, indicating potential mechanisms by which protein malnutrition-induced muscle wasting is counteracted. Additionally, Lp^WJL^ supplementation alleviated the reduction in cortical bone thickness and the deterioration of trabecular bone microstructure in the femur. Taken together, these results indicate that Lp^WJL^ can protect against skeletal muscle atrophy and compromised bone microarchitecture caused by protein malnutrition, providing novel insights into the potential therapeutic applications of probiotics for treating malnutrition-related disorders.

## Introduction

Malnutrition remains a critical global health concern, particularly affecting infants and young children. In 2023, an estimated 149 million children under five years of age suffered from stunted growth, with approximately half of the deaths in this age group attributed to nutrient deficiencies [1–3]. Prolonged malnutrition disrupts cellular metabolism and results in functional impairments across multiple organ systems, including brain development, cardiovascular system, metabolic function, and gastrointestinal tract [4–6]. Furthermore, it compromises immune function, increasing susceptibility to life-threatening infections [7, 8]. Notably, nutritional deficits during critical growth periods, such as early childhood, pregnancy, and lactation, can cause significant weight loss and reduced muscle and bone mass, potentially triggering early-onset sarcopenia and osteoporosis. These conditions are linked to diminished physical ability and decreased quality of life [9–13].

In recent decades, numerous studies have focused on the significant function of the gut microbiota and the mechanisms underlying host-microbe interactions that contribute to health and disease [14–16]. In early life, the gut microbiota affects child growth and development through multifaceted pathways, including immune, metabolic, and endocrine systems [17]. Recent research has revealed that the gut microbiota in malnourished children exhibits reduced diversity and delayed maturation compared with that in their healthy peers [18]. Another study also found that immature microbiota from undernourished children, when transplanted into germ-free mice, resulted in stunted growth, altered bone morphology, and metabolic disorders in muscle, liver, and brain [19]. Although these studies suggest that malnutrition-induced microbiome modification contributes to child growth defects, the exact mechanisms of action of the gut microbiome behind these processes remain to be elucidated. Probiotics are a type of beneficial bacteria within the gut microbiome that help promote gut health. There is growing evidence that probiotics can improve intestinal flora balance, prevent or treat diarrhea, and alleviate symptoms of irritable bowel syndrome through their anti-inflammatory effects and enhancement of immune function [20–22]. Interestingly, the recent application of probiotics has demonstrated significant potential as a safe and effective therapeutic option for addressing the adverse effects of child malnutrition. Previous clinical trials, for example, showed that daily supplementation with the probiotic *Bifidobacterium infantis* EVC001 can enhance weight gain and reduce markers of intestinal inflammation in Bangladeshi infants with severe acute malnutrition [23]. Furthermore, several studies have shown that probiotics such as *Lacticasebacillus rhamnosus* GG and *Limosilactobacillus reuteri* are effective in reducing the severity of infectious diarrhea in malnourished infants, shortening the duration of hospitalization and decreasing the number of episodes of diarrhea [24–26]. Additionally, previous observations using one strain of the bacterium, *Lactobacillus plantarum* strain WJL (Lp^WJL^), discovered that it can stimulate weight gain and longitudinal growth through interactions with the intestinal microbiota and the somatotropic hormone axis under nutritionally deficient conditions in mice and drosophila [27–29].

Although increasing evidence supports that probiotic therapy can promote systemic growth in malnourished conditions in both human and animal models, the mechanisms responsible for these effects, especially how probiotics enhance specific organ functions, remain largely unclear. Therefore, this study aimed to investigate the mitigative effects of oral supplementation with Lp^WJL^, a well-known growth-promoting probiotic strain, on muscle atrophy and bone deterioration caused by protein deficiency in juvenile mice. Our malnutrition mouse model confirmed that early-life protein deficiency leads to impaired muscle and bone growth. Importantly, Lp^WJL^ supplementation significantly attenuated these severe negative consequences by preventing the loss of muscle mass and function and mitigating bone structural deterioration.

## Materials and methods

### Animals

3-week-old male C57BL/6 mice were obtained from Orient Bio Inc. (Gapyeong, South Korea) and housed in a temperature-controlled room (22 ± 2°C) with a 12:12 h light-dark cycle. Mice were randomly assigned into three groups: normal diet (CON, *n* = 25), protein-malnourished diet (P-MAL, *n* = 25), and protein-malnourished diet with *Lactobacillus plantarum* WJL (Lp^WJL^) administration (P-MAL Lp^WJL^, *n* = 25). Body weights were measured to ensure similar distribution among the groups before starting the dietary treatment. The sample size was determined through an a priori calculation using G*Power software and by referencing previous publications with similar experimental conditions [27, 28]. The CON group was given a standard protein diet (28% protein, Altromin 1414, Altromin, Germany) and water *ad libitum*. The P-MAL and P-MAL Lp^WJL^ groups were fed a protein-malnourished diet (9% protein, Altromin C1003, Altromin, Germany), receiving 70% of the CON group’s intake volume, administered at the same time each day. Mice experienced weight loss as a result of the dietary intervention, which was an expected outcome of the experiment. Body weight was measured three times per week on the same days and times to ensure consistency in data collection. To minimize potential bias, at least two researchers were involved at every stage of the experiment. All animal procedures in this study were approved by the Institutional Animal Care and Use Committee (IACUC) at Seoul National University (SNU-191010-2-8) and conducted in accordance with the university’s ethical guidelines for the care and use of laboratory animals.

### Bacterial strain preparation

*Lactobacillus plantarum* strain WJL used in this study were cultured overnight at 37°C in MRS medium containing 4% ethanol up to a concentration of 1.8 x 10^9^ CFU/ml. After centrifugation (6000g for 10 min at room temperature), the supernatant was discarded, and the pellet was then dissolved in 50 ml of autoclaved tap water. Fresh bacterial preparations were prepared daily and administered to the mice through their drinking water.

### Grip strength

The maximal force exerted by the limb muscles was evaluated using a grip strength meter (BIO-GS3, Bioseb, France) following the manufacturer’s instructions. The measurements were performed in a blinded manner. Briefly, mice were allowed to grasp the grid with their whole limbs and were then gently pulled backward by their tails until they released their grip. Five consecutive trials were conducted for each mouse and the maximal grip strength for each trial was recorded. Both the average and the highest values obtained from five trials were used for the analysis.

### Maximal running capacity

Mice were acclimatized to the treadmill (Panlab-Harvard Apparatus, Barcelona, Spain) by running at a speed of 15 cm/sec with a 5° incline for 5 min/day for 3 days. The maximal treadmill test was performed at a fixed incline of 5°, starting with a 5 min warm-up at a speed of 15 cm/sec; the speed was then progressively increased by 3 cm/sec every 5 min, until reaching a running speed of 45 cm/sec. The speed was then maintained until the mice reached exhaustion. Exhaustion was determined when the mice remained in contact with a rear electrical stimulus grid (0.3 mA) for 10 sec. The total time of exercise was recorded and converted to distance.

### Tissue collection and histology analysis

Following 6 weeks of dietary intervention, the mice were weighed, anesthetized with an injection of Avertin (125 mg/kg), and then euthanized by excision of the heart. Tissue collection was conducted after complete euthanasia to minimize pain and distress. Major organs (liver, kidney, spleen, heart and epididymal fat) and skeletal muscles (soleus [SOL], plantaris [PLA], gastrocnemius [GA], tibialis anterior [TA], extensor digitorum longus [EDL], and quadriceps [QUAD]) were then harvested and weighed. The muscle tissues were embedded in Tissue-Tek O.C.T compound (Sakura Finetek #4583), flash-frozen in 2-methylbutane pre-cooled in liquid nitrogen and stored at −80°C. Sections of frozen muscle (10-μm-thick) were air-dried for 10 min, fixed in 4% paraformaldehyde for 5 min, and then washed three times for 5 min each in PBS. The sections were blocked with 3% bovine serum albumin for 1 hr and then incubated with a primary antibody (Rabbit Anti-Laminin, 1:1000, Sigma #L9393) diluted in blocking buffer overnight at 4°C. After washing three times for 5 min in PBS, the sections were incubated with a secondary antibody, Alexa Fluor 488 Goat Anti-Rabbit IgG (1:1000; Invitrogen # A11008) for 30 min. Following another set of three washes for 5 min each in PBS, the sections were mounted with VECTASHIELD antifade mounting medium (H-1000, Vector Laboratories) with a glass coverslip. Images were obtained using a fluorescence microscope (Eclipse Ni, Nikon) at 10X magnification. Muscle fiber cross-sectional area (CSA) was measured using semi-automatic image processing of skeletal muscle histology, a MATLAB application (SMASH) [30].

### Quantitative real-time PCR analysis

Total RNA was extracted from the TA muscle tissues using TRIzol reagent (RiboEx, GeneAll), following the manufacturer’s instructions, and the RNA concentration was determined at 260 nm. The isolated RNA (1 μg) was reverse-transcribed into cDNA using reverse transcriptase 5x buffer, random primers, 2.5 mM dNTP, RNase inhibitor and M-MLV RT. qPCR assays were conducted using SYBR Green Master Mix (SensiFAST™ SYBR Hi-ROX Mix 2x, Bioline) with primer pairs designed to detect different target gene transcripts (Table 1). Relative quantification of gene expression was determined by the comparative cycling threshold analysis method. To determine relative expression levels, target gene expression was normalized to GAPDH and calibrated to the average of the control groups.

**Table 1:** Real-time PCR primer sequences.

Genes	Forward Primer	Reverse Primer
GAPDH	ACCCAGAAGACTGTGGATGG	CACATTGGGGGTAGGAACAC
Atrogin-1	AGGAGCGCCATGGATACTGT	GATGCCACTCAGGGATGTGA
MuRF-1	CATAGCAGAGGCCTTGAGGG	TTTACCCTCTGTGGTCACGC
IGF-1	ACCAAAATGACCGCACCTGC	AACACTCATCCACAATGCCTGTC
GHR	CCTCCATTTGGATACCCTAC	CTCCGTTGTCTGGATCTCAC
SIRT1	ACGGTATCTATGCTCGCCTTG	GACACAGAGACGGCTGGAAC
SOD2	GCCTCCCAGACCTGCCTTAC	GTGGTACTTCTCCTCGGTGGCG

### Western blot analysis

Gastrocnemius muscles were homogenized in RIPA lysis buffer (Biosolution) supplemented with a protease and phosphatase inhibitor cocktail (GenDEPOT). Protein concentration was determined using the Pierce BCA Protein Assay Kit (Thermo Fisher Scientific). Equal amounts of protein (20 μg) were separated on a 10% SDS-PAGE gel and transferred onto nitrocellulose membranes (Bio-Rad). Membranes were blocked with 5% skim milk or 5% bovine serum albumin in 0.1% TBS-T, followed by incubation with primary antibodies: Atrogin-1 (mouse monoclonal, SC #166806, 1:500 dilution), MuRF-1 (mouse monoclonal, SC #398608, 1:500 dilution), P-Akt (rabbit monoclonal, CST #4060, 1:1000 dilution), Akt (rabbit polyclonal, CST # 9272, 1:1000 dilution), P-p70S6K (rabbit polyclonal, CST #9204, 1:1000 dilution), p70S6K (rabbit polyclonal, CST #9202, 1:1000 dilution), and GAPDH (rabbit monoclonal, CST #2118, 1:1000 dilution). The membranes were then incubated with horseradish peroxidase-conjugated secondary antibodies: goat anti-rabbit IgG (CST #7074, 1:2000 dilution) or horse anti-mouse IgG (CST #7076, 1:2000 dilution). When analyzing the same blot with different antibodies, stripping buffer (Biosolution) was used according to the manufacturer’s instructions. After the original primary and secondary antibodies were removed, the blot was blocked with 5% skim milk in 0.1% TBS-T, and re-probed with different antibodies. Immunodetection was performed using enhanced chemiluminescence reagents (EZ-Western Lumi Pico Kit, DoGenBio) and band intensities were quantified using ImageJ software.

### Micro-CT analysis

Femurs were carefully isolated, cleaned of excess soft tissue, and then fixed in 4% paraformaldehyde overnight at 4°C. Following fixation, the bones were rinsed with PBS and stored in 70% ethanol until further analysis. The cortical and trabecular compartments of the femurs were evaluated using a Skyscan 1276 μCT device (Bruker, Kontich, Belgium). For cortical bone analysis, the femur samples were scanned at a voltage of 70 kV and a current of 200 μA, using a pixel size of 16 μm, a rotation step of 0.4°, and frame averaging set to 2. For analysis of trabecular bone, the settings were a pixel size of 8 μm, a rotation step of 0.8°, and frame averaging set to 2. After 3D volume reconstruction with NRecon software, femur length was analyzed with DataViewer (Bruker, Kontich, Belgium) and cortical thickness and trabecular bone parameters were analyzed with CTAn software (Bruker, Kontich, Belgium). Cortical bone thickness was measured at the mid-diaphysis, expanding to a length of 1.1 mm (70 slices). The trabecular region of interest was segmented from the cortical shell for a 1 mm section (165 slices) in a region below the most distal portion of the growth plate for each mouse. The following parameters were assessed for 3D volume: trabecular bone volume fraction, trabecular separation, trabecular number, and trabecular thickness.

### Statistical analysis

All statistical analyses were conducted using GraphPad Prism 9 statistical software. Statistical significance was determined using unpaired two-tailed *t*-tests for CON vs. P-MAL and P-MAL vs. P-MAL Lp^WJL^. Data are presented as means ± SEM. *P*-values < 0.05 were considered statistically significant.

## Results

### Growth-promoting effects of probiotic Lp^WJL^ in a mouse model of malnutrition

**Fig 1:**
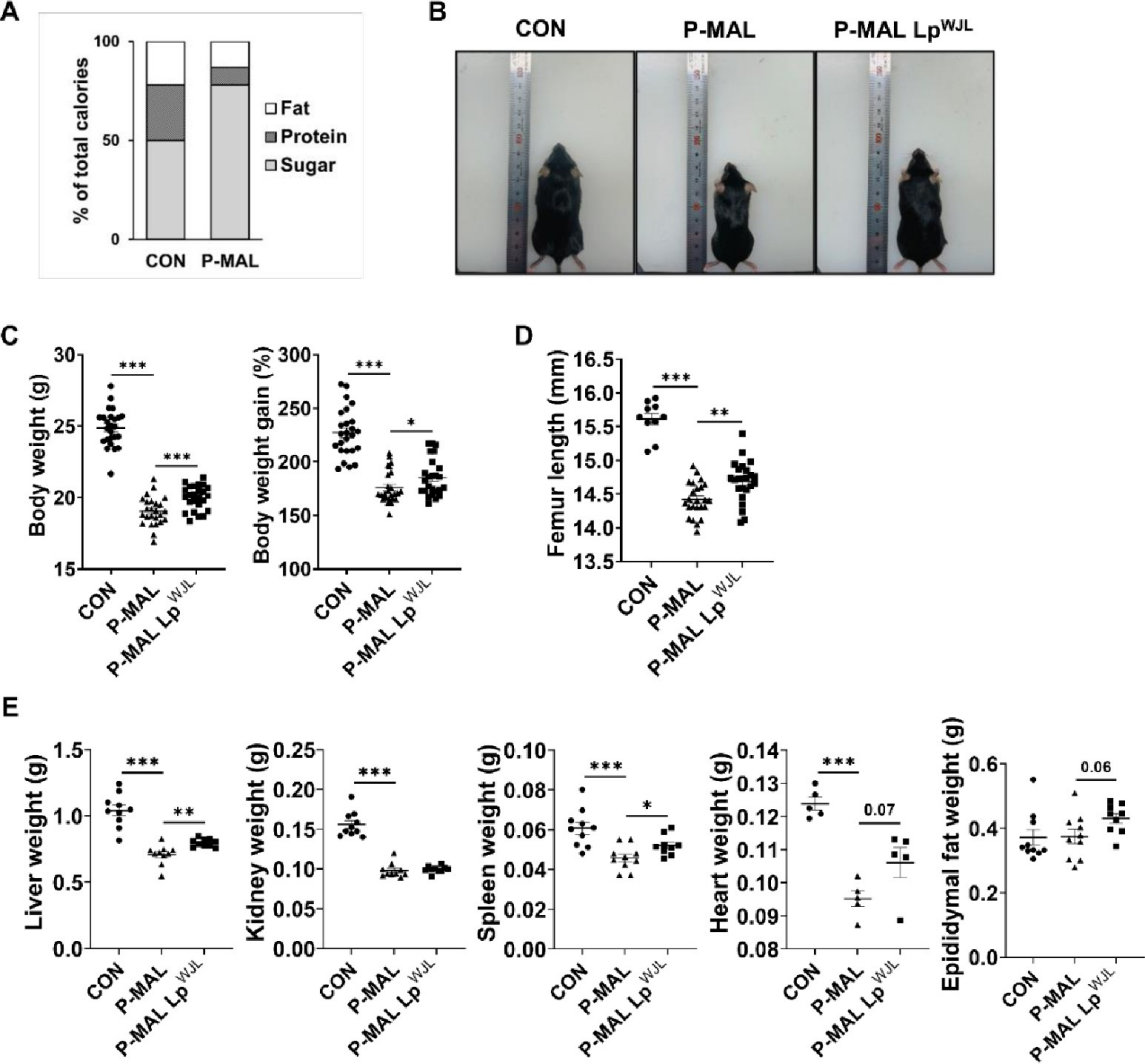
Lp^WJL^ intervention to promote growth and development in juvenile mice with protein malnutrition. (A) A schematic of the nutritional components of the CON and P-MAL diets, expressed as a percentage of total calories. (B) Representative images of C57BL/6 male mice exposed to CON and P-MAL diets with or without probiotic Lp^WJL^ supplementation for 6 weeks. (C) Changes in body weight and body weight gain post-probiotic supplementation (*n* = 25 per group). (D) Femur length after 6 weeks of CON, P-MAL, and P-MAL Lp^WJL^ diet feeding (CON, *n* = 10; P-MAL and P-MAL Lp^WJL^, *n* = 24 per group). (E) Changes in major organ weights (liver, *n* = 10; kidney, *n* = 10; spleen, *n* = 9–10; heart, *n* = 5; and epididymal fat (Epi. fat), *n* = 9–10). Statistical significance was determined using unpaired two-tailed *t*-tests in C, D and E. Data are presented as means ± SEM. *P < 0.05, **P < 0.01, ***P < 0.001.

To confirm the growth-promoting effects of Lp^WJL^ in a juvenile mouse model of protein malnutrition, we initiated the study on postnatal day 21. Mice were assigned to one of three diets: a control diet (CON: 28% protein), protein-malnourished diet (P-MAL: 9% protein), or P-MAL supplemented with Lp^WJL^ (P-MAL Lp^WJL^) (Fig 1A). Both the P-MAL and P-MAL Lp^WJL^ groups received a moderated chow volume, equivalent to 70% of that provided to the control group to establish a protein malnutrition mouse model. In the first week of diet exposure, P-MAL diet-fed mice exhibited significantly lower body weights than those on the CON group. This trend continued, with marked decreases in body weight and weight gain of –23% and –22% after 6 weeks, respectively, compared with the CON group (Fig 1C). However, Lp^WJL^ supplementation mitigated these effects, leading to modest increases in body weight, weight gain, and organ mass, such as the liver and spleen, compared with P-MAL diet alone. Moreover, Lp^WJL^-supplemented mice exhibited longer femur lengths than those fed the P-MAL diet alone, suggesting a modest increase in body height (Fig 1B–1D). These findings indicate that Lp^WJL^ modestly improves overall growth parameters in protein-deficient mice.

### Beneficial role of probiotic Lp^WJL^ on protein malnutrition-induced muscle atrophy

**Fig 2:**
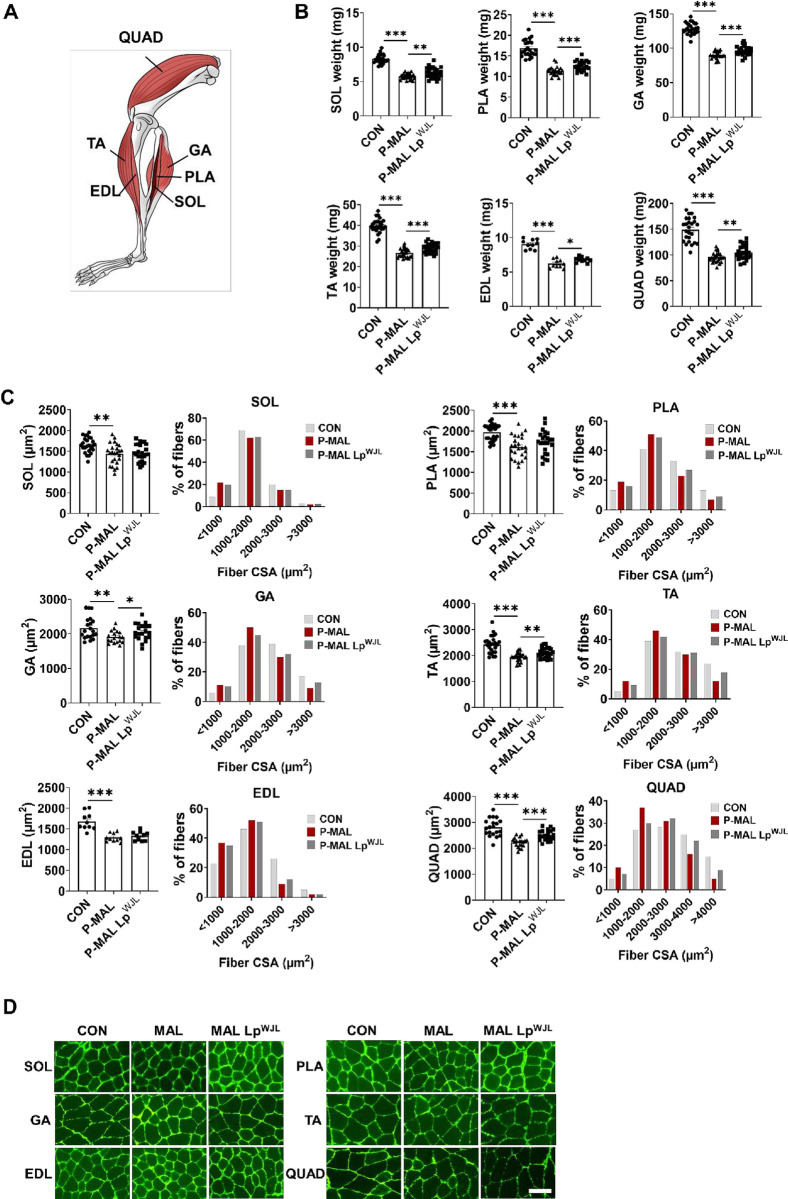
Effects of Lp^WJL^ supplementation on mitigating protein malnutrition-induced muscle loss. (A) Schematic diagram of mouse hindlimb muscles. (B) Weights of the SOL (*n* = 25), PLA (*n* = 24–25), GA (*n* = 25), TA (*n* = 25), EDL (*n* = 10), and QUAD (*n* = 25) muscles after 6 weeks of feeding with CON and P-MAL diets with or without Lp^WJL^ supplementation. (C) Average cross-sectional areas (CSAs) of the SOL (*n* = 25), PLA (*n* = 24–25), GA (*n* = 23–25), TA (*n* = 24–25), EDL (*n* = 10), and QUAD (*n* = 20) muscles from each animal (left) and comparison of frequency histograms displaying the percentage muscle fiber size after CON, P-MAL, and P-MAL Lp^WJL^ diet feeding (right). (D) Representative images of cross-sections from anti-laminin-stained SOL, PLA, GA, TA, EDL, and QUAD muscles after administering the CON, P-MAL, and P-MAL Lp^WJL^ diets. Scale Bar = 100 μm. Statistical significance was determined using unpaired two-tailed *t*-tests in B and C. Data are presented as means ± SEM. *P < 0.05, **P < 0.01, ***P < 0.001.

Maintaining muscle mass and size is essential for muscle strength, functionality, and exercise capability and is also closely linked to overall health, mobility, and quality of life. However, the influence of probiotics on muscle mass and size under protein malnutrition conditions during the growth period remains unknown. Therefore, we investigated the impact of Lp^WJL^ supplementation on preserving muscle mass and size under such conditions. We focused on six different skeletal muscles, including both slow-twitch oxidative fibers (SOL) and fast-twitch glycolytic fibers (specifically PLA, GA, TA, EDL, and QUAD), from mouse hindlimbs (Fig 2A). As shown in Fig 2B, after 6 weeks of P-MAL diet consumption, a significant decline in muscle mass was observed across various muscles (SOL –31%, PLA –32%, GA –30%, TA –33%, EDL –31%, and QUAD –36% *vs*. the CON group). This adverse effect was significantly reversed by Lp^WJL^ supplementation, resulting in substantial increases in muscle mass across the evaluated muscles (SOL 9%, PLA 11%, GA 7%, TA 9%, EDL 9%, and QUAD 10% *vs*. the P-MAL group) (Fig 2B).

Given the beneficial effect of the probiotic Lp^WJL^ on muscle mass in protein-malnourished mice, we further investigated alterations in muscle fiber size. Histological analysis revealed significant reductions in muscle fiber cross-sectional area (CSA) across the six different muscles (SOL –13%, PLA –18%, GA –12%, TA –20%, EDL –23%, and QUAD –20% *vs*. the CON group). However, Lp^WJL^ treatment significantly enlarged muscle fiber size, particularly in the GA, TA, and QUAD muscles (GA 8%, TA 8%, and QUAD 12% *vs*. the P-MAL group) (Fig 2C and 2D). Although the average CSA of the SOL, PLA, and EDL muscle fibers remained unchanged, frequency histograms revealed an increased subpopulation of relatively large fibers (> 2,000 μm^2^) in the PLA and EDL muscles of Lp^WJL^-supplemented mice (Fig 2C). Collectively, these results demonstrate that dietary protein deficiency during growing stages severely affects both slow- and fast-twitch muscle fibers of skeletal muscles, and Lp^WJL^ supplementation effectively mitigates these adverse outcomes by increasing muscle mass and fiber size under protein malnutrition conditions.

**Fig 3:**
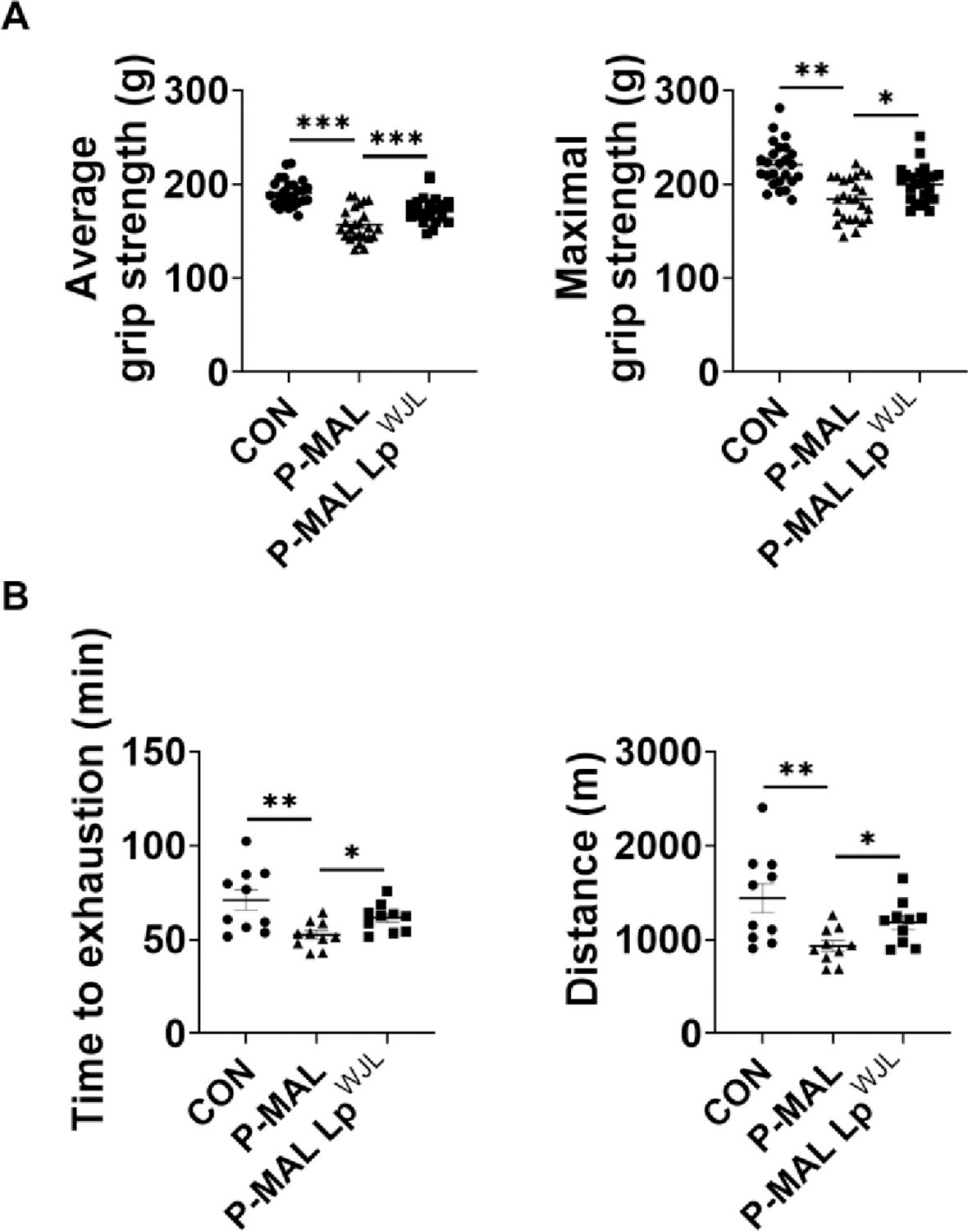
Enhanced muscle strength and endurance exercise capacity via Lp^WJL^ supplementation in dietary protein restriction. (A) Average from five trials (left) and average peak force data (right) from the grip strength test (*n* = 25 per group). (B) Maximal treadmill running time (left) and distance (right) during a graded treadmill speed test after the 6-week intervention (*n* = 10 per group). Statistical significance was determined using unpaired two-tailed *t*-tests. Data are presented as means ± SEM. *P < 0.05, **P < 0.01, ***P < 0.001.

We next performed noninvasive *in vivo* tests, including the limb grip strength and treadmill exhaustion tests, to determine whether probiotic-induced increases in muscle mass and size enhance muscle function. P-MAL diet-fed mice exhibited diminished work capacity, as indicated by decreased muscle strength and shorter running times to exhaustion compared with their CON counterparts. However, the probiotic intervention for 6 weeks increased grip force by 10% and lengthened running time by 17%, indicating improved muscle function and endurance exercise capacity (Fig 3A and 3B). These findings suggest that Lp^WJL^ supplementation-induced increases in skeletal muscle mass and size are directly associated with improvements in muscle function and exercise capacity.

### Lp^WJL^ protects against protein malnutrition-induced muscle loss

**Fig 4:**
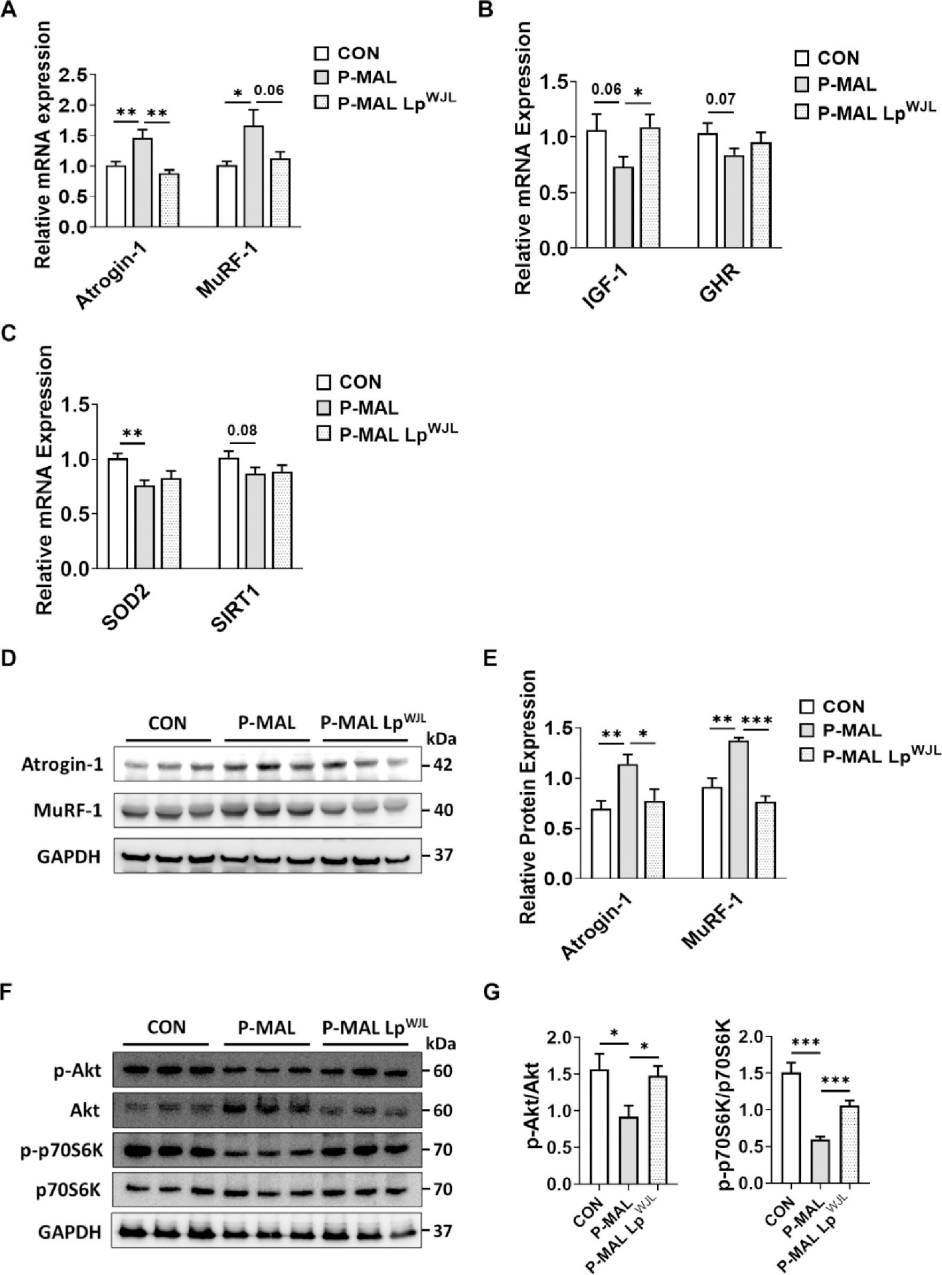
Influence of Lp^WJL^ supplementation on the expression of genes related to muscle growth and loss. (A) mRNA expression analysis of muscle atrophy markers (Atrogin-1 and MuRF-1), (B) growth factors (IGF-1 and GHR), and (C) antioxidant markers (SOD2 and SIRT1) in TA muscles from mice fed CON, P-MAL, and P-MAL Lp^WJL^ diets (*n* = 8–10 per group). (D) Representative western blot images for Atrogin-1, MuRF-1 and GAPDH in GA muscles of mice fed CON, P-MAL, and P-MAL Lp^WJL^ diets. (E) Quantification of Atrogin-1 (*n* = 5–6 per group) and MuRF-1 (*n* = 3 per group) protein levels, normalized to GAPDH levels. (F) Representative western blot images for Akt and p70S6K. (G) Quantification of the phosphorylation-to-total protein ratio for Akt and p70S6K (*n* = 6 per group). GAPDH was used as loading controls. Statistical significance was determined using unpaired two-tailed *t*-tests. Data are presented as means ± SEM. *P < 0.05, **P < 0.01, ***P < 0.001.

Insulin-like growth factor 1 (IGF-1), locally expressed in the muscle, stimulates muscle regeneration and growth by activating the PI3K/Akt/mTOR pathway [31, 32]. In contrast, the expression of genes involved in the protein degradation pathways, such as E3 ubiquitin ligases [e.g., muscle atrophy F-box/Atrogin-1 and muscle-specific RING-finger protein 1 (MuRF-1)], leads to muscle loss [33]. To further elucidate the mechanisms by which Lp^WJL^ intervention ameliorates protein malnutrition-induced muscle loss, we analyzed the expression of genes involved in muscle growth and degradation using qPCR and Western blotting. The P-MAL diet-fed mice exhibited significantly higher expression of Atrogin-1 and MuRF-1 at both the mRNA and protein levels compared to the CON mice (Fig 4A, 4D and 4E). Remarkably, these elevated expressions were almost completely restored to levels similar to those observed in the CON group following Lp^WJL^ supplementation (Fig 4A, 4D and 4E). Furthermore, there was a trend towards decreased expression of genes involved in muscle growth, such as IGF-1 and growth hormone receptor (GHR), during the P-MAL diet. However, Lp^WJL^ supplementation to the P-MAL diet significantly increased the IGF-1 expression to a level comparable to the CON diet group (Fig 4B). Western blot analysis of gastrocnemius muscle revealed that Lp^WJL^ supplementation significantly increased Akt and p70S6K activities, downstream targets of IGF-1 (Fig 4F and 4G). In addition to the protein synthesis and degradation pathways, mitochondrial oxidative stress is an important factor in the loss of muscle mass. We observed a reduction in the mRNA expression levels of mitochondrial superoxide dismutase 2 (SOD2), which plays an important role in protecting against oxidative damage (Fig 4C). Taken together, these results indicate that protein degradation through ubiquitin ligases, alterations in the IGF-1/Akt/p70S6K signal pathway, and mitochondrial oxidative stress, are likely involved in muscle loss in response to the P-MAL regimen, and that Lp^WJL^ supplementation protects against muscle loss, possibly by modulating these pathways.

### Improvement in bone microarchitecture in growing mice via Lp^WJL^ supplementation

**Fig 5:**
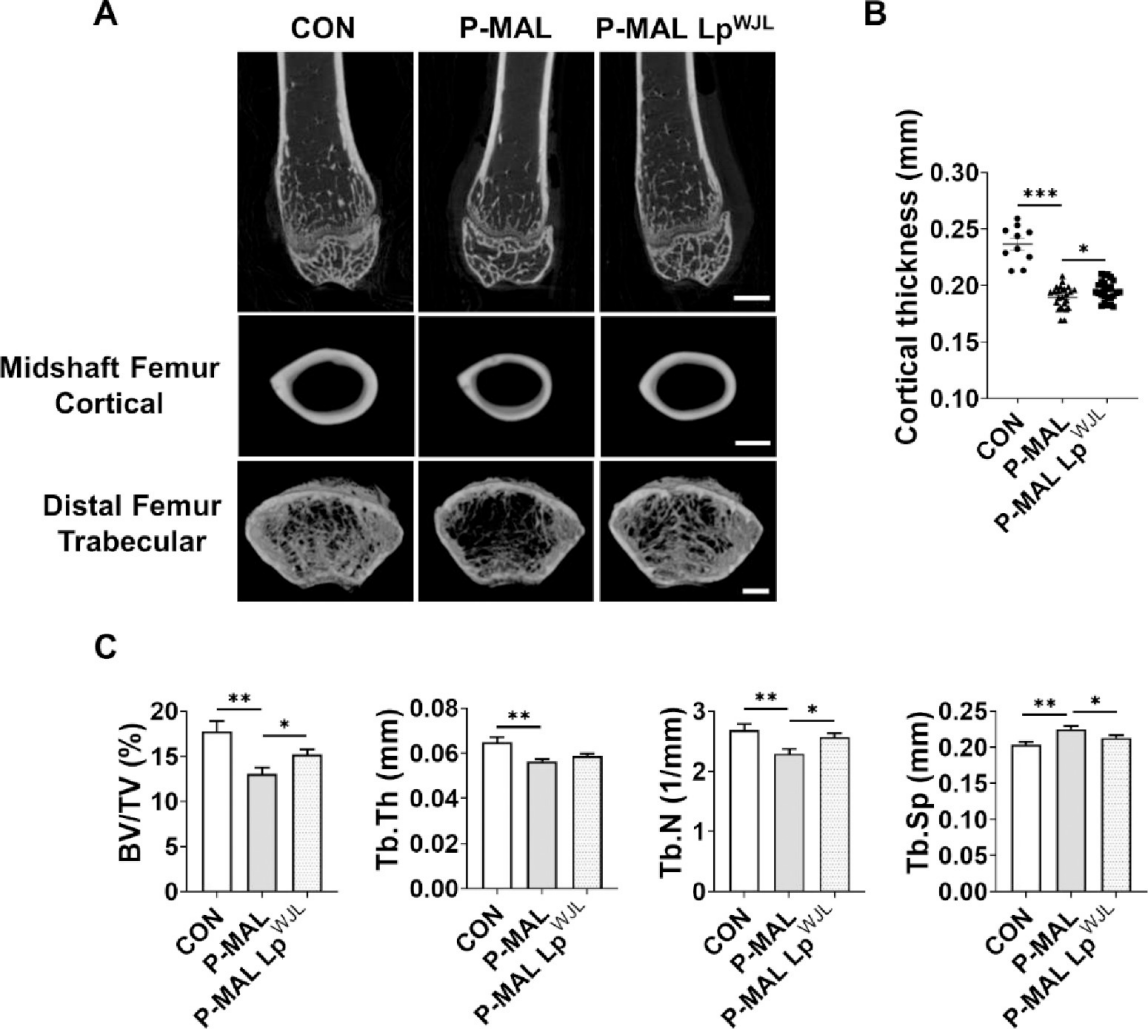
Effects of the Lp^WJL^ probiotic on femur microstructures in protein-deficient mice. (A) Representative 2D and 3D reconstructions using micro-CT images of femurs from mice fed CON, P-MAL, and P-MAL Lp^WJL^ diets. Scale Bar: 1mm for 2D images and 500 μm for 3D images. (B) Alterations in cortical thickness at the femur midshaft in mice fed CON (*n* = 10), P-MAL (*n* = 25), and P-MAL Lp^WJL^ diets via oral administration (*n* = 25) for 6 weeks from postnatal day 21. (C) Trabecular bone parameters at the distal femur metaphysis, including trabecular bone volume fraction (BV/TV, %), trabecular thickness (Tb.Th, mm), trabecular number (Tb.N, 1/mm), and trabecular separation (Tb.Sp, mm), for the CON, P-MAL, and P-MAL Lp^WJL^ groups (*n* = 20 per group). Statistical significance was determined using unpaired two-tailed *t*-tests in B and C. Data are presented as means ± SEM. *P < 0.05, **P < 0.01, ***P < 0.001.

Bone mineral content exponentially increases during childhood, and early bone mass accumulation is crucial for maintaining lifelong skeletal health [34, 35]. Although adequate nutrient intake is vital to supporting optimal bone growth and development during these formative years, few studies have addressed strategies to prevent or treat the detrimental effects of malnutrition on bone health. Therefore, we sought to determine whether dietary protein deficiency induces altered bone morphology and prevents proper bone growth in the femur of rapidly growing juvenile mice, and whether Lp^WJL^ supplementation can mitigate the putative adverse effects caused by malnutrition on the bone microarchitecture. Using micro-CT, we assessed several cortical and trabecular bone parameters, including bone volume-to-total volume ratio (BV/TV), trabecular thickness (Tb.Th), trabecular number (Tb.N), and trabecular separation (Tb.Sp) [36]. Fig 5A displays representative micro-CT images of femoral cortical and trabecular bones after 6-week P-MAL diet feeding, with and without probiotic intervention. Our findings revealed that dietary protein deficiency significantly compromises bone microarchitecture, characterized by a marked reduction in cortical bone thickness (–20%) and adverse changes in trabecular bone structure, including decreased BV/TV, Tb.Th, and Tb.N values (–27%, –13%, and –15%, respectively) and an 11% increase in Tb.Sp in the P-MAL group compared with those in the CON group (Fig 5B and 5C). Importantly, Lp^WJL^ treatment yielded significant improvements in cortical bone thickness and positively influenced trabecular structures, generating increases in BV/TV and Tb.N and a reduction in Tb.Sp (17%, 12%, and –5%, respectively), compared with those of P-MAL group (Fig 5B and 5C). These results indicate that adequate protein intake is required for bone growth, and dietary probiotic intervention alleviates the detrimental effects of protein malnutrition-induced bone loss by enhancing both cortical and trabecular bone structures.

## Discussion

Dietary protein deficiency during growth periods leads to stunted growth with muscle and bone loss. Although additional energy and nutrients are essential, they are often insufficient to completely reverse the detrimental effects of malnutrition. Emerging evidence highlights the benefits of probiotics for enhancing systemic growth in children; however, research into their role as nutritional supplements for addressing the impact of protein malnutrition on various organs is limited. In this study, we demonstrate that daily Lp^WJL^ supplementation can help prevent muscle loss and bone structural deterioration that occur during protein deficiency during critical growth phases.

Skeletal muscle mass and fiber size are remarkably adaptable characteristics that undergo continuous adjustments to regulate muscle strength and metabolism [37, 38]. Based on the myosin heavy chain isoforms they express, muscle fibers are classified into two main types: slow-twitch oxidative fibers, specialized for sustained, low-intensity contractions, and fast-twitch glycolytic fibers, adapted for intermittent, high-intensity contractions [39]. Previous research has reported that malnutrition predominantly affects the mass and size of fast-twitch muscle fibers in adult rat model [13]. Another study found a low-protein diet from lactation to 3 months of age resulted in reduced muscle mass in both slow- and fast-twitch muscle fibers of mice [10]. Given that our results indicate significant growth restriction on both slow- and fast-twitch muscle fiber mass and size in protein-malnourished juvenile mice, we speculate that malnutrition-induced muscle atrophy is more extensive and severe during the growth period. Furthermore, we expanded on previous research by demonstrating the muscle growth capabilities of Lp^WJL^ supplementation, including increased muscle mass and size coupled with enhanced muscle function under conditions of insufficient protein consumption. Notably, Lp^WJL^ supplementation significantly affects the fiber cross-sectional area of fast-twitch muscles, specifically the GA, TA, and QUAD muscles. This pattern suggests a selective responsiveness of different muscle types to probiotic treatment, which may depend on the intrinsic properties of each muscle type.

The skeletal system of a growing body undergoes significant structural adaptations to satisfy increasing physical demands, such as bone elongation, adjustments in mineral composition, and changes in mechanical properties [34, 35]. A previous study highlighted the critical role of nutrition in bone development, indicating that while refeeding after food restriction can initiate growth and bone modeling, it may not entirely reverse the damage caused by prior malnutrition [40]. Our data reinforced the hypothesis that early-life nutrition is vital for proper bone health by demonstrating protein restrictions significantly diminished cortical bone thickness and trabecular structures [41, 42]. However, most of the existing studies have focused on probiotics preventing bone loss during postmenopausal osteoporosis, while little attention has been given to the benefits of probiotics on bone growth and quality during childhood, a critical period for lifelong bone health. Here, our study provides the first evidence that the probiotic Lp^WJL^ can promote longitudinal growth in the long bones and ameliorate the microarchitectural deterioration in both cortical and trabecular bones that occur when consuming a low-protein diet.

Although the exact mechanism by which the bacterium promotes muscle and bone growth during protein malnutrition has not been fully elucidated, prior evidence suggests several possible pathways through which probiotics exert their beneficial effects. Lp^WJL^ is a well-known probiotic strain for promoting postnatal growth. Previous research has identified a mechanism by which Lp^WJL^ alleviates growth delays in undernourished infant mice through nucleotide-binding oligomerization domain-containing protein 2 (NOD2) signaling. Notably, both live bacteria and the membrane fraction or purified peptidoglycan isolated from Lp^WJL^ are sufficient to promote growth by serving as ligands for the host peptidoglycan receptor, NOD2, thereby enhancing intestinal cell proliferation and nutrient absorption [28]. Additionally, Lp^WJL^ administration significantly increases IGF-1 levels in the liver [28]. Interestingly, our study found that Lp^WJL^ supplementation also raises IGF-1 levels in the skeletal muscle of chronically malnourished mice. This suggests that skeletal muscle-derived IGF-1, alongside liver-derived IGF-1, may contribute to the growth-promoting effects of Lp^WJL^. Further research is needed to determine whether the mechanism underlying the increase in skeletal muscle IGF-1 levels with Lp^WJL^ treatment depends on NOD2 signaling.

Lactate, a primary metabolite produced by *Lactobacillus* species, plays a crucial role in muscle metabolism and has been linked to muscle hypertrophy and improved exercise performance. Once considered merely a metabolic by-product, lactate is now recognized as an intercellular signaling molecule that influences muscle function, growth, and repair [43, 44]. Specifically, it activates the mammalian target of rapamycin (mTOR) pathway, which is a central regulator of muscle protein synthesis and growth, while reducing the activity of the ubiquitin–proteasome system involved in muscle protein degradation [45–48]. In the present study, our findings indicate that Lp^WJL^ supplementation suppresses muscle-specific E3 ubiquitin ligases and activates IGF-1 signaling pathways. Further investigation would be valuable to determine whether the increased lactate produced by Lp^WJL^ contributes to the IGF-1 upregulation and Atrogin-1/MuRF-1 downregulation observed in our model.

In addition, probiotics have emerged as a promising treatment for osteoporosis, with studies demonstrating their potential to counteract osteoporosis by modulating immune responses. For example, *Lactobacillus* strains have shown osteo-protective effects on the host immune system via anti-inflammatory and immunomodulatory mechanisms, by balancing regulatory T cells and T helper 17 cells, reducing osteoclastogenic factors (interleukin-6, interleukin-17, tumor necrosis factor-alpha, and RANK ligand), and boosting anti-osteoclastogenic cytokines (interleukin-10 and interferon-gamma) in mice with ovariectomy-induced osteoporosis [49–51]. Given the similar morphological changes between aging-induced and protein malnutrition-induced bone loss, these mechanisms may also be attributed to bone health through Lp^WJL^ treatment; however, future studies are warranted to elucidate the specific mechanisms involved Lp^WJL^-induced bone structural enhancement in malnourished juvenile mice.

This study has several limitations. First, it does not fully elucidate the molecular mechanisms by which Lp^WJL^ modulates IGF-1 and ubiquitin-proteasome signaling. Nuclear factor kappa B and signal transducer and activator of transcription 3 are known to be associated with both IGF-1 and ubiquitin-proteasome signaling pathways [52, 53]. However, our preliminary data indicate that the expression of inflammatory cytokines (e.g., interleukin-6, tumor necrosis factor-alpha, and interleukin-1 beta) remains unchanged under P-MAL conditions or Lp^WJL^ treatment (data not shown). Therefore, at least under our experimental conditions, the inflammatory cytokine signaling pathway does not appear to play a role in regulating IGF-1 and ubiquitin-proteasome signaling. Further studies are needed to clarify the incomplete molecular pathways underlying the beneficial effects of Lp^WJL^. Additionally, it would be valuable to investigate whether Lp^WJL^ acts directly on target organs (i.e., muscle and/or bone) or indirectly through other organ systems or systemic effects to mediate its benefits. Lastly, this study focuses on the restoration of muscle and bone growth in a protein-malnourished juvenile model. Future research is necessary to confirm whether these benefits extend to adult and aged models, particularly in relation to enhancing muscle and bone mass and function during the aging process.

In conclusion, the present study reveals the indispensable role of Lp^WJL^ in mitigating the detrimental effects of a protein-deficient diet on muscle and bone health in juvenile mice. Our findings suggest that protein deficiency, a critical nutritional issue, can severely impact muscle and bone physiology during the growth period. Notably, Lp^WJL^ supplementation is a potentially beneficial therapeutic strategy for effectively preventing malnutrition-induced muscle and bone growth restrictions. Furthermore, given that the harmful effects caused by protein malnutrition resemble those seen in aging-related muscle and bone loss, we can assume that various organ impairments in the aging population may be attributed, at least in part, to a protein deficient-diet. Such insights may inform improved nutritional guidelines for children and the elderly, leading to innovative healthcare solutions that prevent and treat muscle and bone disorders, also potentially addressing aging-related conditions, such as sarcopenia and osteoporosis.

## Supporting information

S1 File Raw data for body and muscle weights. (XLSX)

S2 File Raw data for muscle fiber cross-sectional area (CSA) analysis. (XLSX)

S3 File Raw data for quantitative real-time PCR analysis. (XLSX)

S4 File Raw data for western blot quantification and original images of western blot analysis. (XLSX)

S5 File Raw data for micro-CT analysis of the femur. (XLSX)
